# Microglia and the Blood–Brain Barrier: An External Player in Acute and Chronic Neuroinflammatory Conditions

**DOI:** 10.3390/ijms24119144

**Published:** 2023-05-23

**Authors:** Giorgia Serena Gullotta, Giuseppe Costantino, Maria Angela Sortino, Simona Federica Spampinato

**Affiliations:** 1 Department of Biomedical and Biotechnological Sciences, University of Catania, 95123 Catania, Italy; giorgia.g89@hotmail.it (G.S.G.); giuseppe.costantino@unifg.it (G.C.); 2 Ph.D. Program in Neuroscience and Education, DISTUM, University of Foggia, 71121 Foggia, Italy; 3 Department of Scienza e Tecnologia del Farmaco, University of Turin, 10124 Turin, Italy; simonafederica.spampinato@unito.it

**Keywords:** BBB, endothelial cells, astrocyte, stroke, Alzheimer’s disease, microglia

## Abstract

Microglia are the resident immune cells of the central nervous system that guarantee immune surveillance and exert also a modulating role on neuronal synaptic development and function. Upon injury, microglia get activated and modify their morphology acquiring an ameboid phenotype and pro- or anti-inflammatory features. The active role of microglia in blood–brain barrier (BBB) function and their interaction with different cellular components of the BBB—endothelial cells, astrocytes and pericytes—are described. Here, we report the specific crosstalk of microglia with all the BBB cell types focusing in particular on the involvement of microglia in the modulation of BBB function in neuroinflammatory conditions that occur in conjunction with an acute event, such as a stroke, or in a slow neurodegenerative disease, such as Alzheimer’s disease. The potential of microglia to exert a dual role, either protective or detrimental, depending on disease stages and environmental conditioning factors is also discussed.

## 1. Introduction

The central nervous system (CNS) is surrounded by the blood–brain barrier (BBB), a specialized vascular structure that ensures nourishment, protection and the maintenance of homeostasis. The BBB regulates cerebral blood flow, and consequently the transport of oxygen, glucose and other metabolites, necessary to allow proper CNS functions, and controls the transport of solutes and metabolic waste through the brain vasculature [1]. The BBB is constituted by the endothelial cells (ECs) that form blood vessels and capillaries and their basement membrane. The ECs of the BBB bear structural and functional peculiarities essential to establish a tight control of compounds and cellular trafficking incoming and outgoing from the CNS. The vascular endothelium is indeed characterized by the absence of fenestration and the expression of tight junctions (TJs) that prevent the uncontrolled paracellular passage of water-soluble molecules [2,3,4]. The ECs have also reduced pinocytotic activity, and the passage of nutrients and other compounds occurs only through specific active transport mechanisms.

The endothelium is functionally and anatomically supported by other elements within the CNS that collectively constitute the “Neurovascular Unit” (NVU) [5]: mural cells (pericytes, smooth muscle cells), astrocytes, whose endfeet cover ECs, microglia and neurons. These cellular components are adjuvated by two extracellular matrix structures, recognized as basement membranes (BMs) (named also “basal lamina”). The inner vascular BM surrounds the ECs, separating them from the pericytes, while the outer parenchymal BM is interposed between the pericytes and the astrocytes endfeet [6,7,8]. The BMs play a pivotal role in the maintenance of the close connection between the elements of the NVU [9], provide a reservoir of growth factors and adhesion receptors and contribute to the regulation of the BBB permeability [1,10].

**Figure 1:**
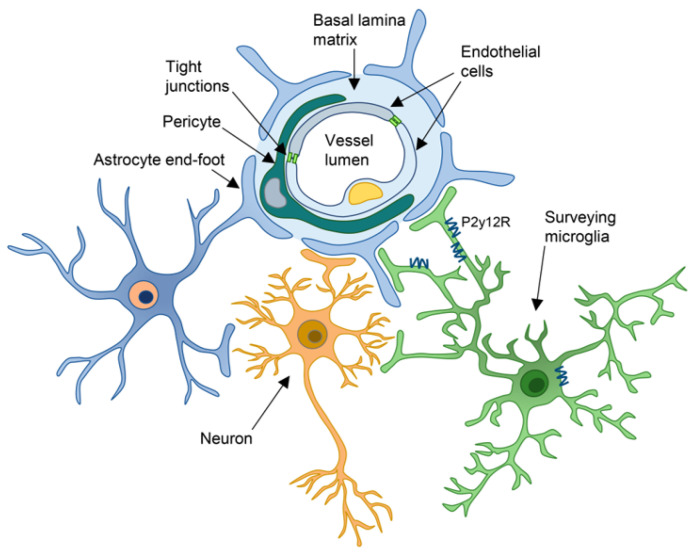
Schematic representation of the Neurovascular Unit (NVU) structure and the reciprocal interaction among all components. The ECs, whose intracellular spaces are sealed by the TJs, are surrounded by mural cells (pericytes or smooth muscle cells). ECs and mural cells are enwrapped by the two basal laminas, which provide structural and functional support to the BBB. The astrocytic endfeet cover almost the whole blood vessel surface and make direct contact with ECs and mural cells. Neurons make contact with ECs and astrocytes, and their activity can regulate the vascular tone either directly on ECs or indirectly through astrocytes. Microglial processes make dynamic and transient contacts with the vasculature, astrocytes and neurons and contribute to the neurovascular coupling through P2Y12R.

Overall, the NVU is a finely organized structure that protects the CNS from toxins, pathogens and injury, thus providing the environmental conditions for a proper neuronal function (Figure 1). Each element of the NVU is structurally and functionally connected to the others, and indeed, the loss or improper activity of one component leads to alterations in BBB properties and can be responsible for CNS disease.

Although much work has been done in the last years to investigate the role of ECs, pericytes and astrocytes in maintaining BBB integrity, the precise contribution of microglia in managing BBB properties in health and disease is still largely undefined. Nevertheless, microglia have recently gained growing attention as a promising therapeutic target in acute CNS injury and in neurodegenerative disorders. As the resident immune cells of the CNS, microglia are extremely sensitive to environmental modifications and rapidly react to external insults in an attempt to re-establish CNS homeostasis. Microglial cells harbor high phenotypic plasticity, and their response to CNS injury is dynamic, so they undergo functional modifications over time during the disease course. Therefore, while reactive microglia in the acute phase of injury can exert a protective function, the persistence of sustained inflammation can switch them toward a detrimental phenotype that compromises neuronal survival and induces BBB damage [11,12]. Of note, under inflammatory conditions causing BBB disruption, microglial cells are able to detect signals from the outside of the CNS and communicate with the peripheral immune players, through the release of soluble mediators. This crosstalk with the periphery influences immune cell trafficking across the BBB and results in a further modulation of the microglial phenotype, eventually affecting neuronal survival and BBB properties. In recent years, the perspective of a beneficial pharmacological modulation of the microglia phenotype has brought the scientific community to shed light on the role of these cells in several pathological settings, either acute or chronic, and on the influence of microglia on BBB integrity, as well as on the interactions with peripheral immunity. In this review, we summarize the current knowledge on the contribution of microglia in the modification of BBB functionality, focusing in particular on changes that can occur following an acute injury, such as a stroke, or in a chronic neurodegenerative disorder that implies a neuroinflammatory state, such as Alzheimer’s disease (AD). We will focus in particular on the interactions between microglial cells and the components of the NVU and on the crosstalk with peripheral immune cells.

## 2. Components of the NVU

### 2.1. Endothelial Cells

Highly specialized ECs are the main cellular component of the BBB. Unlike the endothelium of other body districts, ECs at the BBB are characterized by low transcellular and paracellular permeability, due to the lack of fenestration, the low rate of pinocytosis and the presence of specific junctional proteins: TJs and adherens junctions (AJs) [2]. ECs at the BBB also express very low levels of adhesion molecules, necessary to allow endothelium/leukocyte interaction [13].

TJs and AJs are involved in maintaining junctional integrity. Together, they form a seal between adjacent ECs, limiting the permeability only to small lipophilic molecules and gases. TJs are transmembrane protein complexes and are linked to the cytoskeleton through their intracellular portion [14]. Among other proteins, the most represented are claudin-5, which mostly correlates with TJs functions [15], occludin, an important regulator of TJs functions [14,16], and the intracellular TJ zonula occludens (ZO) family, necessary to connect the transmembrane proteins to the actin cytoskeleton [17,18]. The access into the brain of bigger molecules is strictly dependent on the presence of specific transporters and pumps that mediate the penetration of essential nutrients, such as glucose and amino acids, needed to meet the brain’s energy demands [19]. The passage across the endothelial barrier of nutrients and drugs may be unidirectional, either influx or efflux, or bidirectional, allowing the passage of solutes in both directions, according to the concentration gradient or against it, utilizing the energy of the hydrolytic reaction of ATP [20]. The BBB does not only represent a barrier to solutes, but it can also be considered an immunological barrier, as it prevents the infiltration of antigens and limits the access in the CNS of circulating immune cells, only allowing CNS immunosurveillance [21]. The rate of leukocyte infiltration depends on the expression of AJs, in particular VE-cadherin, and adhesion molecules, such as ICAM-1, VCAM-1 and P- and E-selectins. In physiological conditions, ECs at the BBB express very low levels of leukocyte adhesion molecules [13,22]. Their expression is mainly regulated at transcriptional levels, mostly induced by soluble inflammatory mediators, such as cytokines and chemokines [22]. When highly expressed, adhesion molecules and AJs may be more easily engaged by their leukocyte counterpart, leading to a signaling cascade that terminates with the transendothelial migration and leukocyte access into the CNS. In their role, the ECs are directly supported by astrocytes and pericytes.

### 2.2. Pericytes

Pericytes are contractile cells that enwrap endothelial cells and are responsible for the production of the extracellular matrix and proteins that contribute to the regulation of BBB homeostasis [23,24]. Due to the heterogeneity of mural cells, a clear classification and the nomenclature differentiating pericytes and vascular muscle cells are controversial and beyond the scope of this review [25]. Pericytes are critically required for the establishment of a fully functional BBB, although their absence could not reflect a general loss of BBB markers. It has been recently reported that the junctional protein claudin-5, the glucose transporter Glut-1 and the efflux proteins (P-glycoprotein) were still expressed in animals where pericytes were depleted (Pdgf-b ret/ret). Nevertheless, these animals showed increased BBB leakage, increased ICAM-1 expression and focal leukocyte extravasation [26]. Accordingly, others reported that pericytes control TJs alignment and transcytosis across the BBB [13,27,28]. Pericytes seem to be mostly involved in the regulation of the rapid control of neurovascular coupling. Due to their contractile activity, they can actively relax or contract to modify the cerebral blood flow (CBF) in response to neuronal activity [29,30]. Pericytes are essential in BBB development and in the maintenance of brain microcirculation, as they directly contribute to the formation of the BM [31] and secrete angiogenic-promoting factors (such as VEGF, angiopoietin-1, TGF-β, PDGF-BB) that can support endothelial survival and stabilize BBB functions [32]. A lack of pericytes leads to hyperplasia, abnormal vascular morphogenesis [33] and an incomplete seal of the BBB during embryogenesis, as they inhibit the expression of proteins that increase vascular permeability, such as Angiopoietin 2 and the plasmalemma-vesicle-associated protein (Plvap), and leukocyte adhesion molecules on ECs [34].

Reciprocal interactions exist also between pericytes and astrocytes, and indeed, the absence of pericytes influences astrocyte polarization, thus affecting astrocytic endfeet/endothelial interaction [35]. For their part, astrocytes facilitate pericyte migration, proliferation and contact with the endothelial layer [36,37].

### 2.3. Astrocytes

Astrocyte–endothelial interaction is important for the functions of the BBB. While pericytes are more important in the early stages of BBB property induction, astrocytes are more involved in the maturation and maintenance of barrier properties [38,39], by promoting the expression of intermolecular junctions, enzymatic pathways and specific transporters (e.g., Glut-1) [40]. Several studies have tried to explore how the depletion of astrocytes may affect BBB properties in animal models. Although the laser ablation of a single astrocyte may be not sufficient to induce BBB damage [41], complete astrocyte ablation in adult mice induced an early and sustained BBB dysfunction, due to alterations in TJs functions [42]. Astrocytes are highly secretory cells, and their secretome contains hundreds of molecules [43,44]. For instance, the morphogen sonic hedgehog (Shh) and retinoic acid reinforce endothelial junctional tightness [45,46]. Angiotensinogen, primarily produced by astrocytes [47], is a central player in the regulation of blood pressure, but its role in the promotion or disruption of BBB integrity is controversial: it can both reduce [48] and promote barrier permeability [49]. Astrocytes are the source of the main vascular trophic factor, VEGF-A, promoting angiogenesis and endothelial cell proliferation, differentiation and survival during brain development [50]. In contrast, high levels of VEGF-A may induce BBB disruption in pathological conditions [39]. Astrocytic factors may then both induce and disrupt barrier properties, depending on the disease context, age and surrounding microenvironment. Astrocytes physically contact endothelial cells; astrocytic terminal processes, called endfeet, cover the outer surface of the endothelium and enwrap the vasculature [51]. Endfeet are thought to cover up to 99% of the cerebrovascular surface [52], participate in the regulation of angiogenesis and the formation of cell-to-cell junctions [53] and mediate neurovascular coupling, according to local demands. It has been described that astrocytic endfeet removal, due to invading glioma cells in an animal model, resulted in increased barrier permeability and significant TJs loss [54]. Accordingly, the focal loss of a single astrocyte induced a plastic response in the surrounding astrocytes to maintain vascular coverage [55].

Finally, reactive astrocytes in inflammatory conditions can form their own junctions to limit leukocyte infiltration into the CNS, supplying the BBB barrier when endothelial functions are compromised [56].

### 2.4. Microglia

Microglia are the resident immune cells of the CNS and in the healthy brain and spinal cord represent 10–15% of all cells [57]. They were described for the first time by the neuroanatomist Pio Del Rio-Hortega in 1919, who also noticed the mesodermal origin and phagocytic capability of these cells [58]. In the last decade, a number of elegant studies in mice confirmed that microglia are mesodermal-derived cells, originating from erythromyeloid progenitors of the yolk-sac, which begin to colonize the developing CNS before the formation of the cerebrovascular network, from embryonic day 8.5 until the complete development of the BBB [59,60]. Similarly, microglial precursors in humans infiltrate the CNS primordium starting at around 4.5 gestational weeks (see [61] for review). Progenitors’ differentiation toward microglia requires two transcription factors, Pu.1 and Irf8, whose expression distinguishes microglia from other tissue-resident macrophages [62,63]. In the adult brain, microglia maintenance depends on the constant activation on their surface of Colony-stimulating Factor-1 Receptor (CSF-1R) by interleukin (IL)-34 and CSF-1, which are continuously released by neurons and astrocytes, respectively. The pharmacological blockade of CSF-1R leads indeed to rapid microglia depletion [64,65]. Microglia are long-living and self-renewing cells that under inflammatory conditions can undergo fast proliferation through a mechanism of selected clonal expansion [66]. Nevertheless, in the case of BBB damage in CNS diseases, circulating monocytes can infiltrate the CNS and acquire a microglial-like phenotype [67]. In the healthy brain and spinal cord, microglia harbor a ramified morphology with long and motile processes that continuously scan the surrounding environment [68] accomplishing multiple and different functions in the CNS, along with immune surveillance. In physiological conditions, microglia activity is essential for proper synaptic pruning during development [69,70], synaptic plasticity [71,72,73], neuronal programmed cell death and neurogenesis in adults [74,75], as well as learning and memory processes [76], which are fulfilled through the phagocytosis of excess synapses, neurites and neurons.

Following environmental perturbations or injury, microglia acquire an ameboid morphology, with much shorter ramifications and larger cell bodies [77]. In pathological conditions, microglia employ phagocytosis to clear the parenchyma from protein aggregates, e.g., amyloid-β (Aβ) [78,79], pathogens [80], immune cells that have crossed the BBB and damaged and even stressed-but-viable neurons in a process named “phagoptosis” [81]. Primed microglial cells are able to release a plethora of pro-inflammatory cytokines (IL-1α and IL-1β, Tumor Necrosis Factor (TNF) α), chemokines (CCL2, CCL5, CXCL1, MIP-1), proteases (e.g., matrix metalloproteases, MMPs) and reactive oxygen species (ROS), which, while orchestrating the immunological response to injury, can compromise BBB integrity if their secretion is protracted for a long time. Nevertheless, microglial cells are also able to secrete molecules that promote tissue healing and quench inflammation (e.g., VEGF, TGFβ-1, IL-10) [82,83]. Thus, over the years, it has been increasingly evident that microglia harbor high phenotypic plasticity with activity modifications over time during disease. Starting from the early 2000s, their functional status has been classified as pro- or anti-inflammatory, named M1 and M2, respectively [84]. These terms were originally borrowed from in vitro studies of T-helper cells that, after the administration of specific stimuli, acquired a pro- (Th1) or anti- (Th2) inflammatory activity [84]. Similarly, microglia have been divided into these two categories on the basis of the expression of specific markers that indirectly implied a detrimental (M1) or protective (M2) phenotype [84]. At present, the use of single-cell-transcriptomic and proteomic techniques demonstrated that the M1/M2 definition is too simplistic for microglia, whose dynamic behavior is strongly dependent on the pathological and environmental conditions [84].

The possible influence of microglial cells on physiological BBB functions has been only recently explored. During embryogenesis, microglial cells appear to be closely associated with the developing blood vessels in the retina and throughout neurogenesis in the cortex and actively contribute to vascular development [85]. The absence of microglia in PU.1^-/-^ mice or its pharmacological depletion with the CSF-1R inhibitor PLX5622 results in choroidal vascular atrophy, the disorganization and dysfunction of retinal pigment epithelial cells and an altered expression of angiogenic growth factors [86,87]. Similarly, Csf1^op/op^ mice, which produce a mutated inactive form of CSF-1, show an early post-natal defective retinal vasculature, with altered arterial–venous patterning and reduced branching [88].

In the adult CNS, microglia make transient and dynamic physical contact with the neuro-vasculature, and accordingly, a specific microglial population has recently been described, called capillary-associated microglia (CAM), that interacts with microvessels more stably [89]. Nevertheless, the existence of a “juxtavascular” microglial population was noticed earlier [90]. CAMs represent about 30% of the whole microglial population in adult mice and localize preferentially along the vessels where astrocyte endfeet coverage is absent, raising further investigations on their direct contribution to the regulation of microvessel diameter and, in consequence, of CBF [89,91]. It was shown that microglia depletion through the CSF-1R inhibitor PLX3397 results in an increased capillary diameter by 15% [89], and a recent study by Csaszar and colleagues demonstrated that microglia make simultaneous contact with vascular elements (ECs, mural cells and astrocytes) and neurons to regulate neurovascular responses [92]. This study showed also that microglia have a direct effect on the regulation of the CBF and, in particular, they contribute to neurovascular coupling via purinergic signaling through the receptor P2Y12R. After common carotid artery occlusion, microglia depletion impaired CBF recovery, and its activity was mediated by P2Y12R signaling. Furthermore, it has been demonstrated in vitro that the presence of microglia in co-culture with ECs promotes the barrier properties in the endothelium, inducing the expression of TJs, ZO-1 and occludin proteins [93].

Overall, microglia activity in the healthy CNS contributes to the maintenance of a protected and well-functioning microenvironment, on the one hand, ensuring proper neuronal activity and, on the other hand, interacting with the other components of the NVU to regulate BBB properties.

In the next sections, we recapitulate the current scientific knowledge on the molecular mechanisms of microglia response to acute CNS injury, either acute or chronic, the consequent modifications of the interactions with the elements of the BBB and their influence on BBB properties.

## 3. Acute Injury: How Microglia Affect BBB Properties in Ischemic Stroke

Ischemic stroke is a devastating disease caused by the severe reduction or interruption of the CBF to the brain and is the second leading cause of death worldwide [94]. Stroke involves the disruption of the BBB and neuroinflammatory processes and is one of the main acute disease models in which microglia response and interaction with the NVU components have been investigated.

The evolution of a stroke can be divided into three main phases, characterized by distinct pathophysiological events: the hyperacute phase, the acute phase, which can be further divided into subacute and acute, and the chronic phase [95,96]. The hyperacute phase occurs within the first minutes subsequent to blood flow impairment and lasts for about 6 h. It is characterized by cell death in the ischemic core due to metabolic failure and the priming of microglia and astrocytes. The second phase is characterized by the expansion of the ischemic volume and the possible conversion of the ischemic penumbra into an infarcted core and by the establishment of neuroinflammatory processes [97]. It lasts from 6 h after the onset to the following 72–96 h, depending on the severity of the occlusion and the span of the ischemic event [95,96]. The chronic phase lasts from several days to weeks from stroke onset. At this stage, secondary injury mechanisms and reparative processes co-exist. Tissue damage due to delayed cell death, edema, glial scar formation and inflammation are still present [98], while neurogenesis, synaptogenesis and angiogenesis are activated [99]. Microglia undergo rapid activation following ischemia and start to proliferate 4–6 h after stroke onset, reaching a peak at around 4–5 days to decrease thereafter [100,101]. During the disease course, there have been observed discrete microglia populations harboring pro-inflammatory or protective activity [12], which favor tissue healing through the removal of debris, neovascularization and neurogenesis. These latter processes start during the subacute phase, around 72 h after a stroke, and persist during the chronic phase of the disease [12,96].

**Figure 2:**
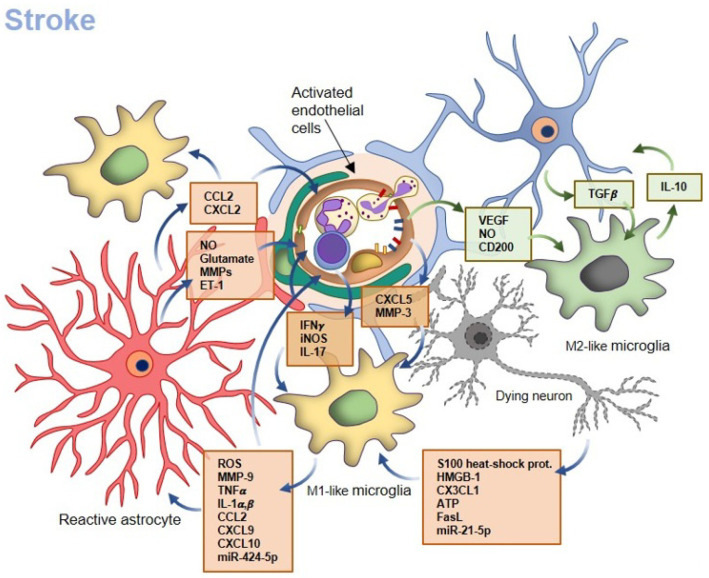
Microglia and BBB function during stroke. During the hyperacute and acute phases of stroke, the damaged/dying neuronal cells release several soluble mediators (reported in the orange box) that drive microglia toward a pro-inflammatory M1-like phenotype. Such primed microglial cells release ROS, MMPs, miRNAs-charged EVs and pro-inflammatory cytokines that activate ECs and astrocytes. These latter, in turn, secrete other mediators (NO, glutamate, endothelin-1 (ET-1)) that further promote EC activation, TJs degradation and BBB disruption. Reactive astrocytes release further pro-inflammatory cytokines (e.g., CCL2, CXCL2) that promote the pro-inflammatory activity of microglia. Activated ECs express several adhesion molecules for leukocytes and secrete CXCL5 and MMP-3 that foster the pro-inflammatory status of microglia. In the chronic phase of stroke, ECs and astrocytes promote a beneficial anti-inflammatory M2-like phenotype in microglia, through the secretion of VEGF and TGFβ, respectively. The release of TGFβ from astrocytes is stimulated by IL-10 derived from M2-like microglia, in a positive feedback loop. In physiological conditions, ECs limit microglia priming through the expression of NO and CD200, to protect BBB integrity.

In this pathological context, the by-directional communication between microglia and the other NVU components, as well as the infiltrating immune cells, influences microglial activity which, in turn, affects BBB functionality (Figure 2).

### 3.1. Microglia–Neuron Crosstalk

In the hyperacute phase, the sudden reduction in blood supply to the brain causes a depletion of glucose and oxygen that leads to a metabolic switch toward anaerobic glycolysis, with the production of lactic acid, a consequent pH decrease and a reduction in ATP. A lack of ATP drives the failure of ionic pumps, with an efflux of K^+^ and a reciprocal influx of Na^+^ that result in cell swelling, termed cytotoxic edema, and induce apoptosis. Injured neurons release key damage-associated molecular pattern (DAMP) molecules that include S100 heat-shock proteins [102], high-mobility group box-1 (HMGB-1) [103], ATP, uric acid, nucleic acids [104] and fractalkine (CX3CL1) [105], which are responsible for the initial activation and polarization of microglia following ischemia [106]. In particular, the expression of CX3CL1 by damaged neurons drives the expression of an “eat-me” signal recognized by microglial cells expressing complement 3a receptor (C3aR) that engulf dying but still viable neurons, exacerbating neuronal loss [107]. Moreover, it has been shown in vivo and in vitro that murine neurons release soluble FasL at 24 h and 72 h after a stroke, activating in microglia the JAK/STAT3 and NF-κB signaling pathways, thus inducing an (M1-like) pro-inflammatory phenotype [108]. Neurons and glia communicate also through extracellular vesicles (EVs), and, accordingly, it has been observed that exosomes derived from scratch-injured PC12 neurons contain miR-21-5p, which exacerbates in microglia the release of pro-inflammatory factors and promotes a neurotoxic phenotype [109].

### 3.2. Microglia–Endothelial Cell Crosstalk

Microglial cells activated by neuronal death release ROS, such as superoxide anions, and other mediators, such as MMPs and inflammatory cytokines and chemokines (TNFα, IL1α, IL1β, Macrophage Inflammatory Protein-1α (MIP-1α)/CCL3, CCL2 and CXCL10), that induce endothelial activation and BBB disruption, which is therefore secondary to microglial priming and occurs within minutes to hours from the ischemic onset [96,110].

Specifically, TNFα reduces claudin-5 and ZO-1 expression on ECs, thus increasing BBB permeability [111], and can induce EC necroptosis [112]. IL-1 activates the ECs that, in turn, express pro-inflammatory cytokines and release MMP-9 that digests the TJs and the ECM proteins, further contributing to BBB leakage [113,114]. Microglia-derived CCL2 and CXCL10 further exacerbate BBB disruption and simultaneously foster peripheral immune cell entrance into the CNS [12]. Barrier permeability can be induced even by the shuttling of specific miRNAs through microglia-derived EVs. A recent study by Xie et al. [115] reported that microglial cells activated by oxygen and glucose deprivation (OGD) release exosomes containing miR-424-5p which causes endothelial permeability.

Notably, in an in vivo model of vascular damage induced by methamphetamine, it has been demonstrated that activated ECs are able to directly drive microglia priming, even in the absence of neurodegeneration [116].

Once activated, ECs expose on their luminal surface leukocyte adhesion molecules, such as P- and E-selectins, ICAM-1 and VCAM-1, which promote leukocyte infiltration into the lesion and secrete several soluble mediators that further contribute to microglia priming. For instance, the endothelial-derived CXCL5 is increased 24 h after a stroke in the cerebrospinal fluid of patients, and its pharmacological blockade significantly reduces microglia activation [117]. Moreover, the release of MMP-3 from ECs was found to activate microglial cells and promote inflammation in a mouse model of spinal cord injury [118].

Nevertheless, ECs are also able to inhibit the pro-inflammatory phenotype in microglia during the chronic phase of a stroke. The secretion of VEGF by ECs has been demonstrated to be beneficial both in vivo in rats subjected to middle cerebral artery occlusion (MCAO) and in vitro, since it significantly reduces the expression of pro-inflammatory cytokines and iNOS in microglia [119]. Interestingly, even unchallenged ECs have a dampening effect on microglia activation, in order to reduce the risk of unwanted BBB permeability. For instance, endothelial-derived NO and the immunosuppressor molecule CD200, exposed on the surface of brain ECs, are known to suppress microglial activation ad exert an anti-inflammatory action [120,121].

### 3.3. Microglia–Astrocyte Crosstalk

Besides the reciprocal interaction with neurons and ECs, the crosstalk with astrocytes can also modify the microglial phenotype and affect BBB properties. IL-1α, TNFα and complement component 1 (C1q), secreted by activated microglia, drive astrocytes to acquire a detrimental pro-inflammatory phenotype that induces neuronal and oligodendrocyte death [122,123] and directly contribute to BBB breakdown. Reactive astrocytes, indeed, can release molecules that increase BBB permeability such as MMPs [124], nitric oxide (NO) [125], glutamate, which stimulates the N-methyl- D-aspartate (NMDA) receptors on ECs inducing BBB leakage [126], and endothelin-1 [127]. Moreover, activated astrocytes amplify the pro-inflammatory activity of microglia in a positive feedback loop, thus contributing indirectly to the BBB disruption. Indeed, microglia-derived pro-inflammatory mediators, such as IL-1β [128], induce in astrocytes the down-regulation of protective molecules, such as Shh, and increase the production of chemokines, such as CCL20, CXCL2 and CCL2, which are also chemoattractant molecules for peripheral leukocytes [129]. An in vitro study demonstrated that CCL2 secreted from TNFα-primed astrocytes enhanced the pro-inflammatory phenotype of microglia [130]. In addition, astrocyte-derived CCL7 has been shown to induce microglia activation and pro-inflammatory activity both in vitro and in a mouse model of traumatic brain injury [131].

Nevertheless, astrocytes can also contribute to the dampening of the detrimental response and induce a reparative phenotype in microglia. In mixed co-cultures, astrocytes promote an (M2-like) anti-inflammatory phenotype in microglial cells [132]. Previously, Min and colleagues demonstrated that an astrocyte culture-conditioned medium suppresses the IFNγ-induced inflammatory response in microglial cells by increasing the activity of hemeoxygenase-1 [133].

It is worth noting that the synergic activity of neurons and astrocytes in co-culture with microglia activates the expression of microglia-signature genes that are usually lost in vitro, as well as in aging and disease in vivo, and suppresses infection- and injury-associated gene sets. The presence of neurons and astrocytes also promotes a resting ramified morphology and limits the phagocytic capacity in microglia through, at least in part, TGFβ2 signaling [134]. Moreover, IL-10 secretion by microglia stimulates astrocytes to produce TGFβ, which mitigates the microglial pro-inflammatory activity, triggering negative feedback [135].

### 3.4. Microglia–Peripheral Leukocyte Crosstalk

Finally, microglia are able to promote the infiltration of peripheral immune cells into the injured CNS both directly, through the secretion of chemoattractant chemokines (such as CXCL2, CCL2, CXCL8, CXCL9, CXCL10), or indirectly, inducing endothelial activation. Leukocytes can further modulate the microglial phenotype and contribute to the modifications of BBB properties and eventually the disease outcome. Neutrophils are among the first cells to invade the ischemic lesion, and like microglial cells, they can exert a dual role, either detrimental or beneficial. Neutrophil invasion starts around 3 h after a stroke and peaks at around 24 h, then slightly diminishes in the next seven days [136]. It has been widely demonstrated that neutrophil infiltration is associated with ischemic damage worsening, through the phenomena of vessel occlusion and in particular no reflow and micro-thrombosis, the damage of the endothelial walls and consequent increased BBB leakage [137]. In particular, specific neutrophil subpopulations endowed with high detrimental pro-thrombotic and pro-inflammatory activity have been identified [138]. Nevertheless, microglia actively engulf viable and apoptotic neutrophils, as demonstrated in vitro, in brain slices [139] and also in vivo, a process that is essential to limit tissue damage. Neutrophil phagocytosis occurs mainly at the periphery of the ischemic lesion, where microglial cells are viable and more reactive, while in the ischemic core, local microglial loss and dystrophy lead to the accumulation of neutrophils. Of note, prolonged microglia depletion causes increased neutrophil infiltration and enlarged ischemic lesions [140]. A recent study [141] showed that in systemic inflammation induced by LPS, neutrophils actively interact with microglial processes, probably inducing activation, and then exhibit reverse transmigration to the bloodstream, potentially propagating the inflammatory signals.

During cerebral ischemia, other leukocyte populations enter the lesion site and shape microglial function. For instance, NK cells, which are attracted by microglia-derived CXCL10 and accumulate into the CNS from 3 h after a stroke [142], are able to kill selectively resting microglial cells [143]. Alternatively, microglia secrete IL-15 which enhances the activation of NK cells, promoting BBB disruption in ischemic stroke [144]. The release of CXCL9 and CXCL10 from microglia attracts also CD4^+^ T helper 1 (Th1) lymphocytes, which contribute to the inflammatory response through the secretion of pro-inflammatory cytokines, such as IFNγ and iNOS, thus exacerbating tissue injury directly and indirectly, through microglial activation [145,146]. Similarly, Th17 cells promote microglial pro-inflammatory activity through IL-17 secretion [147], while microglia stimulate Th17 cell differentiation through IL-23 and IL-6 [148]. Th2 and T-regulatory cells (Tregs) instead inhibit inflammation. It has been demonstrated that Tregs depletion after MCAO markedly increases ischemic volume and microglial cell numbers, most of which produce TNFα, highlighting the importance of crosstalk between T cells and microglia [149]. Anti-inflammatory cytokines and chemokines, such as IL-4, CCL17, CCL22 and CCL24 produced by protective microglia, promote the recruitment and differentiation of Th2 lymphocytes that amplify tissue healing [150].

## 4. Microglia in a Chronic Inflammatory Condition: Alzheimer’s Disease

### 4.1. The Impairment of Endothelial Cells at the BBB in Alzheimer’s Disease

Alzheimer’s disease (AD) is the principal cause of memory loss and cognitive decline worldwide. Aβ plaques and hyperphosphorylated tau tangles are pathognomonic hallmarks of the disease, leading to neuronal degeneration, synaptic loss and gliosis.

Neurons are the main target in AD. The reduction in synaptic functions and neuronal degeneration is responsible for the progressive cognitive decline and memory impairment. Aβ can directly act on neurons, where it modifies the activity of NMDA glutamate receptors, NA/K ATPases, insulin receptors and integrins [51,151,152,153,154] and can directly modify neuronal membrane structure, altering its permeability and excitability [155]. In addition, Aβ oligomers can be internalized in neurons, directly contributing to the generation of ROS [156,157].

Nevertheless, BBB impairment has been proposed as one of the key elements in the pathogenesis of AD. Cerebrovascular diseases, responsible for altered vascular perfusion, cerebral hemorrhage and hypoxia, can be common coexisting comorbidities with AD, often representing the first event (first hit) leading to progressive Aβ overload (second hit) [158,159]. A compromised BBB, associated with increased permeability, can be regarded as a biomarker of the normal aging process [160]. In AD patients, damage of the BBB has been shown even in the early stages of the disease: imaging studies and postmortem tissues indicate the leakage of blood-borne proteins and immune cells through the BBB in several brain areas, including the hippocampus, entorhinal cortex and prefrontal cortex [161,162,163]. Accordingly, brain ECs derived from induced pluripotent stem cells of AD patients displayed altered properties, such as a dysregulated expression of TJ proteins and enhanced barrier permeability [164]. Increased interaction between neuronal and non-neuronal cells, in particular among neurons, ECs, astrocytes and microglia, is often observed in AD postmortem brain by immunohistochemistry [165]. In AD, the modification occurring at the BBB exacerbates Aβ accumulation in the brain. As previously mentioned, ECs at the BBB express specific transporters that regulate the movement of molecules between the brain and the blood [166]. Two of these transporters, the Receptor for Advanced Glycated End products (RAGE) and the low-density lipoprotein receptor-related protein 1 (LRP-1), bind and transport Aβ [167]. Their activity is dysregulated in AD [168,169], leading to Aβ accumulation in the brain [166]. The expression of glucose transporter Glut-1 is also severely affected in microvessels in the brain of AD patients and mouse models [170,171], inducing early endothelial dysfunction and barrier failure [172]. Finally, the neurovascular uncoupling, described in the disease, leads to increased permeability to blood-borne solutes and to immune cells, both driving a CNS inflammatory response [173].

Aβ itself may be directly responsible for the modifications described: in in vitro BBB models, EC exposure to Aβ oligomers was responsible for TJs redistribution [174,175] and increased the expression of the adhesion molecules ICAM-1 and VCAM-1 [176,177,178]. Although conflicting results on animal models exist [14], several human postmortem studies indicate that the levels of occludin, ZO-1 and claudin-5 are reduced in the cerebral blood vessels of AD patients [179,180]. Impairment of BBB junctions, which may be directly correlated not just with the decreased levels of the proteins but also with the disruption of the balance among all junctional proteins [181], can facilitate the access of plasma proteins that exacerbate inflammation and neurotoxicity [182]. Finally, the endothelial overexpression of adhesion molecules such as ICAM-1 and VCAM-1 facilitates leukocyte transmigration, leading to the aggravation of the inflammatory response. Accordingly, preventing the access of neutrophils to the CNS has been associated with enhanced cognitive performance and diminished AD-related pathology in AD mouse models [183].

**Figure 3:**
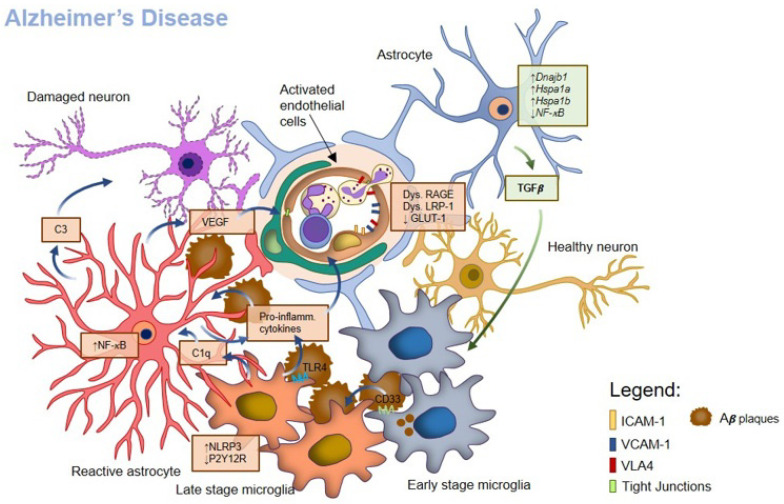
Time-dependent role of microglia in BBB function during development of AD. In the early stage of AD, microglial cells surround and engulf Aβ aggregates to limit their deposition. At later stages, Aβ accumulate in the brain and, by binding to CD33 receptor on microglia, reduce its clearance. Activation of microglia for prolonged periods leads to the secretion of pro-inflammatory cytokines and C1q that drives astrocyte activation and the release of C3, responsible for neuronal and EC damage. In addition, dysregulation of RAGE and LRP-1 and down-regulation of GLUT-1 in ECs lead to Aβ accumulation. Aβ aggregates can also bind to TLR4 on microglial surface and stimulate the NLRP3 inflammasome. Microglia-derived IL-1β stimulates astrocytes to release VEGF, which lead to the degradation of TJ proteins and BBB permeability. Endfeet of astrocytes surrounding Aβ plaques bear structural alterations. In the early stage of AD, astrocytes support the BBB, through the activation of heat-shock signaling (*Dnajb1*, *Hspa1a*, *Hspa1b*) and the down-regulation of NF-κB. Protective astrocytes release TGF β, which induce a protective phenotype in microglia.

BBB properties are strongly affected by endothelial interaction with the other components of the NVU, in particular with glial cells (Figure 3). In AD, glial cells may both directly and indirectly participate in neuronal loss and in the setup of neuroinflammation, a condition that also influences BBB properties.

### 4.2. Microglia Role in AD

Microglia, as the immune competent cells of the CNS, are the first to be activated and attracted by Aβ plaques. Large-scale genome-wide association studies in AD mouse models identified in microglia and myeloid cells more than 20 loci associated with risk factors for the disease [184]. Microglia may start to be involved even a decade before the onset of clinical symptoms, as soon as Aβ plaques start accumulating [185]. In mouse models and AD patients, microglia colocalize with Aβ deposits and, in the early stages of the disease, may create a sort of putative barrier limiting further deposition and plaque-associated neuritic dystrophy [186,187], actively participating in the disassembly and digestion of senile plaques [188]. The use of genetically modified mouse models demonstrated that microglia release complement factors C1q and C3 that are involved in Aβ homeostasis [189]; accordingly, C3R ablation in APP mice resulted in reduced Aβ accumulation [190].

The triggering receptor expressed on myeloid cells 2 (TREM2) expressed by microglia is also involved in Aβ degradation [191]. Interestingly, TREM2 has a disease-progression-dependent effect on Aβ deposition, and although data correlating TREM2 deficiency and/or mutations with Aβ burden have often been controversial [192], several pieces of evidence point out that, at least in the early phases of the disease, TREM2-expressing microglia promote Aβ degradation and clearance and reduce neurodegeneration [155,193,194,195]

However, in the long run, microglial roles may change, triggering the release of pro-inflammatory cytokines and promoting neurodegeneration [155], which can be, in fact, the consequence of Aβ-induced reactivity on glial cells. In particular, abnormal microglial phagocytosis has been described in AD [189]. As mentioned, synaptic pruning is a mechanism undertaken by microglia and is essential to eliminate weak or unnecessary synapses. Microglial phagocytosis is regulated via several mechanisms, including the CX3CR1/CX3CL1 interaction and the complement. Alterations in the complement pathway are described already in the early stages of the disease [189]. Aβ *per se* in fact can bind and activate the complement system [196] and induce the microglia release of the C1p complement factor, which increases the astrocytic release of C3, finally leading to synaptic phagocytosis [189,197]. Accordingly, the levels of C1q and C3 were found upregulated in an AD mouse model, and the deficiency of C1q, C3 and its receptor C3R rescued synaptic density [189].

A constant microglial activation results in the upregulation of inflammatory mediators and cytokines, leading to significant neuroinflammation and neuronal death [155,198]. Aβ may directly bind to the microglial TLR4 receptor [199] and increase the stimulation of the NLRP3 inflammasome [200], leading to the activation of caspase-1 and the consequent cleavage and secretion of IL-1β and IL-18 [201,202]. An increased amount of caspase 1 was reported in the hippocampus and frontal cortex of AD and MCI patients [203]. Accordingly, the deficiency of both NLRP3 and IL-1β protects mice models from spatial memory impairment and improves neuronal functions, while reducing Aβ load in the CNS due to microglial phagocytosis [203]. Furthermore, by binding to the reactive microglial CD33 receptor, Aβ reduces its own clearance, and, accordingly, knocking out CD33 in APP/PS1 mice attenuated Aβ plaque deposition and cognitive decline [204,205].

Besides the interaction with neurons, microglia also exert a dual role in the modulation of BBB properties in AD. Being activated by a constant inflammatory stimulus, microglia can indeed contribute to BBB leakage [206,207,208]; however, at least in the early phases of the insult, in AD, as well as in other CNS pathological conditions, microglia can support the BBB [11,209]. In an in vitro model, the presence of microglia reduced endothelial permeability induced by an inflammatory stimulus [209]. Accordingly, in a mouse model, reactive microglia migrated toward the endothelial barrier, promoting the expression of the endothelial junctional protein, claudin-5, thus supporting ECs in reducing BBB leakiness [11]. In a 5x FAD AD mouse model, selective microglia depletion during the period of plaque setup increased vascular hemorrhage and Aβ deposition in blood vessels, supporting the protective role of microglia on vasculature [210]. Furthermore, the neurovascular coupling mediated by microglia through the purinergic receptor P2Y12R is impaired in AD, thus affecting the cerebrovascular response to neuronal activity and reducing endothelial barrier properties [92,211].

### 4.3. Astrocyte Role in AD and Their Crosstalk with Microglia

Of note, the crosstalk between microglia and astrocytes is affected in AD [82]. Microglia are more sensitive to Aβ and are often responsible for the astrocytic switch toward a more inflammatory-prone phenotype [122,212]. Aβ induces the microglial release of complement factors and cytokines, which affect astrocytic response. The C1q complement component, released by reactive microglia, induces the astrocytic release of the C3 complement factor [122], which is responsible for neuronal damage, and affects EC functions at the BBB [213]. In AD animal models, microglia release TNFα and TGF-β that modulate astrocytic calcium-ion-dependent signaling and glutamate release [214], responsible for the onset of excitotoxicity [215]. In rats, IL-1β, released by microglia, induced the astrocytic release of VEGF [216], while in in vitro studies, IL-1β, derived from co-cultured microglia, suppressed the astrocytic release of Shh [128], a factor essential for endothelial properties at the BBB. Finally, microglia-derived TNFα and IL-1β suppress the expression of astrocytic gap junctions, inducing an altered astrocytic communication, thus facilitating the reactive gliosis observed in AD [217].

Reactive astrocytes are usually found in proximity to Aβ plaques [218], and atrophic astrocytes have been described in postmortem AD [219] and all conditions that lead to the loss of astrocytic homeostatic functions and increased BBB damage [220]. Astrocytes are involved in the generation and clearance of Aβ [221,222,223], although human-iPSC-derived astrocytes and mouse astrocytes over-expressing APOE4 are less efficient in this regard [224,225].

In AD patients and mice models, astrocytic endfeet that surround vascular Aβ deposits show structural alterations, swelling retraction and separation [221], leading to BBB damage. The accumulation of Aβ in the brain triggers the NF-κB pathway in astrocytes, leading to the complement activation and the release of inflammatory cytokines, chemokines and factors that modify neuron–glia communication and synaptic transmission and globally contribute to the onset of neuroinflammation and BBB damage [122,226,227,228]. Together with inflammatory cytokines, in vitro Aβ exposure induced astrocytes to release VEGF, leading to the degradation of the junctional protein claudin-5 and endothelial barrier permeability [229]. Accordingly, in postmortem brain tissues from AD patients, an increased number of newborn immature vessels has been described, as a consequence of increased VEGF levels. The newborn vessels are more permeable, a condition that compromises BBB integrity [160].

Depending on the disease stage, astrocytes may also exert a protective role, cooperating in the elimination of neuritic dystrophies in AD mouse models and in AD patients [230] and releasing factors, such as TGF-β, that increase microglial phagocytic activity and reduce neuronal damage [230,231].

In addition, astrocytes may also support BBB properties in AD. In response to exogenous stressors, such as Aβ accumulation, astrocytes activate heat-shock response, such as *Dnajb1*, *Hspa1a* and *Hspa1b* [232], which restore their ability to maintain BBB functions [233]. The upregulation of the gene *Nr4a1* prevents astrocyte inflammatory response, by reducing NF-κB activity, and contributes to the maintenance of BBB integrity [234]. Astrocytes also modulate leukocytes’ access to the perivascular space [56]. They can, for instance, upregulate the release of protease inhibitors, such as *Pcsk1n*, to protect the endothelial barrier against the degradative enzymes released by infiltrating leukocytes [234]. At least after a short Aβ exposure in vitro, astrocytes mediate the endothelial expression of the adhesion molecule ICAM-1, preventing leukocyte transmigration [229]. In contrast, when the barrier is heavily affected, astrocytes may directly interact with infiltrated leukocytes, modulating their functions. Indeed, lymphocytes exposed to Aβ in the presence of astrocytes were skewed toward a Th2 phenotype, thus increasing the release of BDNF and IL-4 that reduced endothelial barrier damage [235].

However, as already mentioned, in the long run, leukocyte infiltration may cooperate in the setup of neuroinflammation, promoting cognitive impairment, so the blockade of leukocyte transmigration may represent a possible therapeutic approach [207,236].

### 4.4. Pericyte Crosstalk with Microglia in AD

After Aβ exposure, microglia can finally induce pericyte loss [173,237]. In diseases such as AD, pericyte dysfunction can also participate in BBB disruption. Pericyte loss has been described in both AD patients and mice models [238,239], due to Aβ-induced toxicity [240]. In AD patients, the diameter of small capillaries, in particular in the proximity of senile plaques, are narrowed and irregular [241], and a recent study suggested the involvement of contracted pericytes as a causative element [242]. The loss of pericytes may also upregulate the expression of VEGF, contributing to barrier leakiness [33]. Furthermore, Aβ-activated microglia may induce the pericyte release of MMP9 [243] which, as already mentioned, is one of the principal causes of TJs degradation and BBB leakiness [229].

## 5. Conclusions

The BBB is essential to maintain the homeostasis of the CNS. The ECs are functionally supported by the elements of the NVU. This complex structure has multiple key roles: it not only regulates the blood flow and the exchange of molecules and/or cells coming into and out of the CNS but also covers a pivotal role during disease, orchestrating the neuroinflammatory response. Microglial cells have gained particular attention in the last decades, as an increasing number of varied functions have been ascribed to these cells. Microglia are indeed directly involved in the regulation of neuronal activity and turnover, in the immune surveillance and even in the regulation of the CBF. Notably, the association with blood vessels also allows microglia to scan the compounds or cells as soon as they enter the CNS and rapidly react in case of danger. Large scientific evidence has underlined in several pathological conditions the dual behavior of microglia, which is beneficial in the early phase of the disease in the attempt to re-establish homeostasis but turns detrimental when the noxious stimuli persist in the long period. Neurodegenerative disorders and aging are an example of how the microglia phenotype becomes dysfunctional over long time periods, having failed to repair the tissue damage. Even in an acute condition such as a stroke, the persistence of activated microglia up to one year after the onset can cause neurodegeneration [244]. The phenotypical changes of microglia are evident also in short time windows, as described by Haruwaka and colleagues: at 4–7 days after LPS administration, microglia phagocytose the astrocytic endfeet, thus promoting BBB disruption [11]. Similarly, in the subacute and chronic phase of ischemic stroke, the presence of highly pro-oxidant M1-like microglia aggravates neuronal death and disease outcome [129].

The elements of the NVU are functionally connected to each other and trigger positive or negative feedback mechanisms to initiate and subsequently dampen the inflammatory response, in order to recover homeostasis. Microglia actively interact with each element of the NVU and with peripheral immune players, integrate signals and shape accordingly their phenotype to influence the BBB functions and ultimately the disease outcome. Thus, the pharmacological modulation of the microglial phenotype seems to be a promising therapeutic strategy for several neurological diseases. However, recent experimental evidence showed that there is still a gap of knowledge of the interactions between microglia and the vascular elements and the shades of phenotypical modifications, which are highly time- and disease-dependent. Therefore, each new discovery opens new questions on microglia’s biology, which is still a challenging open field of investigation.

## Data Availability

Not applicable.
